# *In Silico*-Motivated Discovery of Novel Potent Glycogen Synthase-3 Inhibitors: 1-(Alkyl/arylamino)-3H-naphtho[1,2,3-de]quinoline-2,7-dione Identified as a Scaffold for Kinase Inhibitor Development

**DOI:** 10.3390/ph16050661

**Published:** 2023-04-28

**Authors:** Thomas D. Emmerich, Joseph M. Hayes

**Affiliations:** School of Pharmacy & Biomedical Sciences, University of Central Lancashire, Preston PR1 2HE, UK

**Keywords:** AKT2, CNS-active, GSK-3α, GSK-3β, *in silico*, inhibitors, kinase selectivity

## Abstract

Glycogen synthase kinase-3 (GSK-3) isoforms α and β have diverse roles within cell biology, and have been linked with multiple diseases that include prominent CNS conditions such as Alzheimer’s disease and several psychiatric disorders. In this study, motivated by computation, we aimed to identify novel ATP-binding site inhibitors of GSK-3 with CNS-active potential. A ligand screening (docking) protocol against GSK-3β was first optimized, employing an active/decoy benchmarking set, with the final protocol selected based on statistical performance analysis. The optimized protocol involved pre-filtering of ligands using a three-point 3D-pharmacophore, followed by Glide-SP docking applying hinge region hydrogen bonding constraints. Using this approach, the Biogenic subset of the ZINC15 compound database was screened, focused on compounds with potential for CNS-activity. Twelve compounds (*generation I*) were selected for experimental validation using *in vitro* GSK-3β binding assays. Two hit compounds, **1** and **2**, with 6-amino-7H-benzo[e]perimidin-7-one and 1-(phenylamino)-3H-naphtho[1,2,3-de]quinoline-2,7-dione type scaffolds were identified with IC_50_ values of 1.63 µM and 20.55 µM, respectively. Ten analogues of **2** (*generation II*) were selected for structure activity relationship (SAR) analysis and revealed four low micromolar inhibitors (<10 µM), with **19** (IC_50_ = 4.1 µM)~five times more potent than initial hit compound **2**. Selectivity screening of low micromolar inhibitors **14** and **19** (comparing aryl- and alkyl-substituents) against 10 homologous kinases revealed unique selectivity profiles, with both compounds more potent against the GSK-3α isoform (IC_50_s~2 µM) and, additionally, inhibitors of PKBβ (IC_50_s < 25 µM). Compound **14** also inhibited ERK2 and **19**, PKCγ, but generally good selectivity for GSK-3 isoforms over the other kinases was observed. The compounds had excellent predicted oral bioavailability and CNS-activity profiles, presenting promising candidates for future testing in cellular models of disease.

## 1. Introduction

Glycogen synthase kinase-3 (GSK-3) is a critical serine/threonine kinase that phosphorylates numerous proteins [1]. The enzyme was first identified as a regulator of glycogen metabolism, phosphorylating and inhibiting glycogen synthase [2]. However, GSK-3 is now recognised as a critical component in many cellular processes [3,4], and its dysfunction implicated in many major diseases [5]. The hypothesis that GSK-3 can be exploited as a target for therapeutic benefit is therefore supported, with aberrant GSK-3 activity linked with conditions including type-2 diabetes, cancer, inflammation, and numerous neurodegenerative and psychiatric disorders [5,6,7,8,9,10]. With respect to the latter, GSK-3 has attracted considerable interest for the discovery of new chemical agents against CNS conditions such as Alzheimer’s disease, epilepsy, schizophrenia, and bipolar disorder [5,7,8,9,10]. Research has also shown that GSK-3 inhibition can be an effective treatment route for different cancer types [11,12]. Some of the pathways that GSK-3 regulates, modulates, or activates are tied to chemo-/radiotherapy resistance and onco-gene transcription [13]. Specifically, through interaction with associated signalling pathways, GSK-3 is linked to the transcription of genes which increase cancer’s ability to survive through down-regulation of apoptosis, as well as through increases in angiogenesis, mobility, and proliferation [14,15]. Targeting GSK-3 has shown potential for cancers including breast, colon, and ovarian, as well as brain cancers such as glioblastoma [12,16,17].

GSK-3 (E.C. = 2.7.11.26) exists in two isoforms, GSK-3α (483 amino acids) and GSK-3β (420 amino acids), that share 85% sequence homology overall, and 97% homology in the kinase domain [18]. The major structural differences between isoforms are at the N- and C-terminals, with GSK-3α having an N-terminal glycine-rich extension. The GSK-3 isoforms have differences in tissue distribution, their cellular localization, and functions, but both GSK-3α and GSK-3β isoforms are enriched in brain tissue [16,19]. GSK-3α is especially abundant in the hippocampus, cerebral cortex, striatum, and cerebellum, while GSK-3β is more widely expressed in nearly all brain regions [19,20]. Significantly, it has been demonstrated in the brain that GSK-3 isoform-specific genetic deletion resulted in distinct changes in substrate phosphorylation, an indication that the isoforms have some differences in their range of substrates [21].

**Figure 1:**
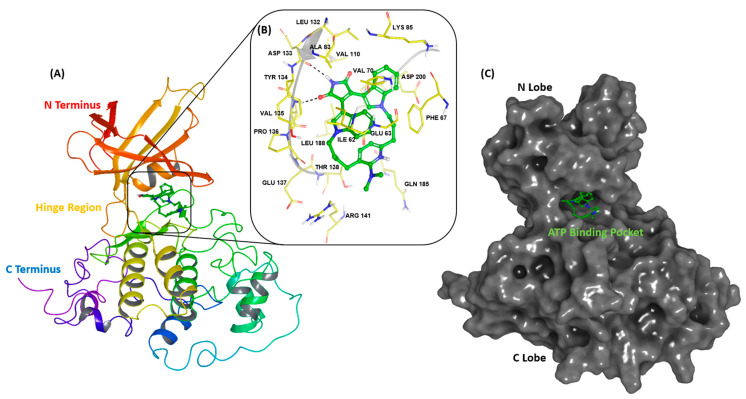
The solved structure of GSK-3β in complex with bis-(indole) maleimide pyridinophane (PDB ID: 2OW3). (**A**) Ribbon representation of the tertiary structure showing the GSK-3β domains: a C-terminus α-helical domain and N-terminus β-sheet domain. (**B**) Binding of the native ligand at the ATP-binding site. Hydrogen bonds are shown as dashed black lines. The key hinge region residues are Asp133-Val135. Protein–ligand hydrogen bonds to Asp133 O and Val135 NH for this inhibitor were observed, but Val135 O also has strong hydrogen bonding potential. (**C**) Protein surface representation, highlighting the native bis-(indole)maleimide pyridinophane inhibitor bound at the ATP-binding site.

While many studies have been performed to design GSK-3 inhibitors, no candidate has yet resulted in an approved drug to treat a particular condition. However, several GSK-3 inhibitors have reached preclinical and/or clinical development [12,22,23]. The main reason for lack of GSK-3 inhibitor progression to the clinic has been in part linked to selectivity issues and off-target effects, so that the design of other structurally-diverse and novel chemical entities with potentially better therapeutic outcomes is needed. Few studies have specifically looked at compounds that have CNS-active profiles at the initial *in silico* screening stage of inhibitor discovery, which is prioritized in this work. The majority of research has focussed on the GSK-3β isoform and ATP-binding site inhibitors; GSK-3β is very well structurally characterized, while the structure of GSK-3α remains unsolved. Inhibitors binding at the ATP-binding site exploit favourable interactions in the so-called hinge region, which, in the case of GSK-3β, are residues Asp133-Val135, as well as interactions with the key binding site residues lining the site (Figure 1) [24,25]. A number of co-crystallized complexes of GSK-3β with different inhibitors have been solved and deposited in the RDCB PDB database (www.rcsb.org (accessed on 1 April 2023)), and have revealed significant protein structure flexibility on inhibitor binding. Considering this, an extensive docking study comparing 13 solved GSK-3β protein–ligand structures for discovery of ATP-binding site inhibitors demonstrated that the more open cavity of PDB ID: 2OW3 with a bound bis-(indole)maleimide pyridinophane inhibitor (Figure 1) was superior to the other solved PDB structures in retrieving known inhibitors from large decoy datasets [26].

Within this study, with a focus on identifying compounds with CNS potential, we first designed and optimized an *in silico* screening protocol (benchmarking studies) for the recognition of known structurally-diverse potent ATP-binding site inhibitors. This was then applied to identify novel inhibitors within the biogenic subset (which includes both primary metabolites and secondary metabolites (natural products)) of the ZINC15 database [27]. Potential for blood–brain barrier permeability was considered throughout, using relevant properties from the QikProp ADME prediction program [28]. The benchmarking protocol used Glide docking [28] with an active/decoy ligand set, with performance for recognition of actives (the known inhibitors) analysed using statistics. Pre-docking pharmacophore filtering was found to considerably improve performance. Following subsequent screening of the biogenic database using the optimised protocol, candidate GSK-3β inhibitors (*generation I* set of compounds) were selected from the predictions and validated as true inhibitors using *in vitro* GSK-3β binding assay experiments. A *generation II* set of compounds based on the 1-(phenylamino)-3H-naphtho[1,2,3-de]quinoline-2,7-dione hit scaffold resulted in a number of potent low micromolar inhibitors, and allowed for a structure activity relationship (SAR) analysis. Tautomeric features of a prototype inhibitor were explored using DFT calculations. Finally, two potent *generation II* analogues were screened *in vitro* against a panel of ten other homologous kinases to determine their kinase selectivity profiles. For this, we were also interested in determining the GSK-3α/β isoform preferences, given the potential significance for different diseases [7].

## 2. Results and Discussion

### 2.1. Design of In Silico Screening Protocol/Benchmarking Studies

**Figure 2:**
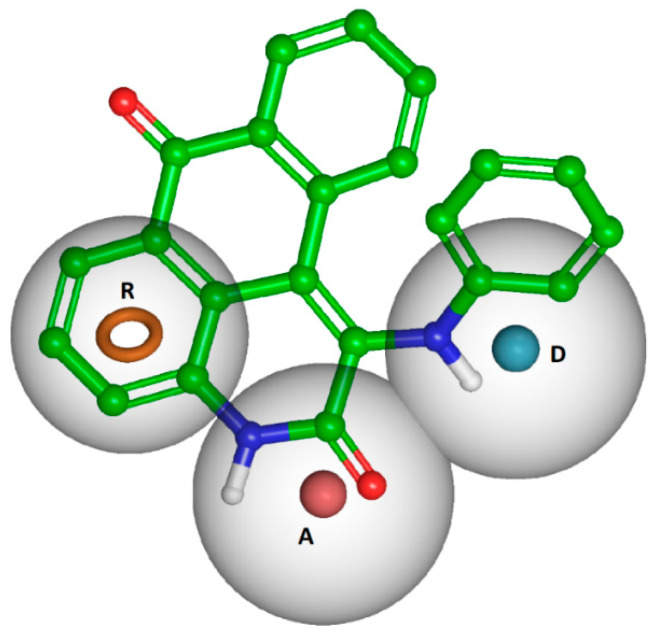
The three-point 3D-pharmacophore hypothesis with ADR features (Table S2) which was used as the pre-docking filter for active compounds in screening ***protocol 3***, as described in the text. The pharmacophore is shown with initial hit compound **2**. (A = hydrogen bond acceptor; D = hydrogen bond donor; R = aromatic group).

The PDB ID: 2OW3 inhibitor-bound GSK-3β complex (Figure 1) was selected for calculations based on its previous performance in docking studies [26]. To create a validated screening protocol, a representative group of 50 known ‘active’ ligands (inhibitors with IC_50_s ≤ 500 nM, Table S1) was used to assess different docking approaches with the program Glide [28]. Statistical analysis of performance was measured in terms of the ability of the docking approaches to recognise the actives among a set of generated decoys (3050) and 142 real inactives (compounds with IC_50_s > 100 µM), the results of which are shown in Table 1. The docking approaches considered were three-fold. The initial approach used Glide-SP (standard precision) docking without any geometric binding constraints (***protocol 1***). In ***protocol 2***, the effect of applying hinge region protein–ligand hydrogen bonds as docking constraints was considered, as these interactions are generally crucial to good ATP-binding site inhibitors; constraints have previously been proposed to reduce false-positive predictions in kinase virtual screens [29]. More specifically, ***protocol 2*** used ligand hydrogen bond constraints on Asp133 O and Val135 O and NH backbone atoms, with two of the three constraints required to be fulfilled. The last approach (***protocol 3***) applied a pre-filtering 3D-pharmacophore prior to docking using the aforementioned hydrogen bond constraints of ***protocol 2***. The Phase v 6.7 pharmacophore was designed considering both the active and inactive compounds, the results of which are shown in Table S2. The pharmacophore model selected (Figure 2) was the highest-ranking hypothesis that contained both hydrogen bond acceptor and donor as features (necessary for hinge region protein–ligand hydrogen bonds); the third feature identified as important for activity was an aromatic group. The pharmacophore also fit the native ligand of PDB ID: 2OW3. Only three-point 3D-pharmacophores were considered, as inclusion of further features would limit diversity of selection in the final screening protocol.

Analysis of the statistical performance results in Table 1 for ***protocols 1***–***3*** shows that there is clear improvement in statistics for recognition of actives going from ***protocol* 1** to ***protocol 2*** to ***protocol 3***, with the combination of pre-docking 3D-pharmacophore filtering and docking hydrogen bonding constraints clearly improving performance. While the area under the ROC curve (AU-ROC) statistic, representing the probability an active is ranked higher than an inactive, is slightly lower for ***protocol 2*** (0.76) and ***protocol 3*** (0.77) compared to ***protocol 1*** (0.80), this is simply due to a small number of the actives (~8–9 of the 50) being filtered out. The constraints and pharmacophore filtering (***protocol 3***) resulted in the best statistical values for all other parameters. Of particular interest is recovery of actives in the top 1% of database (top-32 ligands for benchmarking set) and for this we see, for example, that the efficiency factor (EF—1%) for ***protocol 3*** (34) was better than ***protocol 2*** (28), and more than two times better than ***protocol 1*** (16), which applied no constraints. ***Protocol 3*** was therefore 34 times better than random selection for identification of actives (19 actives were recovered in the top-32 ligands), highlighting the benefits of explicit consideration of hinge region hydrogen bonds, but also the positional presence of an aromatic group in the active compounds (Figure 2). The enhanced enrichment effects are also observed for the top-2% of database (top-ranked 65 ligands), although not to the same degree as for top-1%. EF—2% had values of 15, 16, and 22 for ***protocols 1***, ***2*** and **3**, respectively; there was a similar trend for top-5% of database (top-ranked 162 ligands), where enrichment statistics were again improved with ***protocol 3***, but much less compared to what was observed for the top-1% and -2% of database. Overall, therefore, it was considered that screening of the biogenic database using ***protocol 3*** could be pursued with confidence, focussing on selection of compounds that had the highest predicted ranks.

### 2.2. Screening of Biogenic Database

#### 2.2.1. *Generation I* Selection

**Scheme 1:**
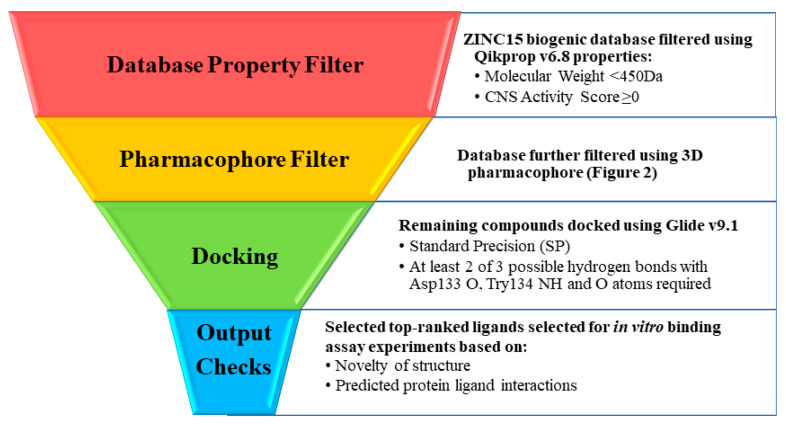
A workflow overview of ***protocol 3*** used for virtual screening and selection of *generation I* compounds for experimental validation.

**Figure 3:**
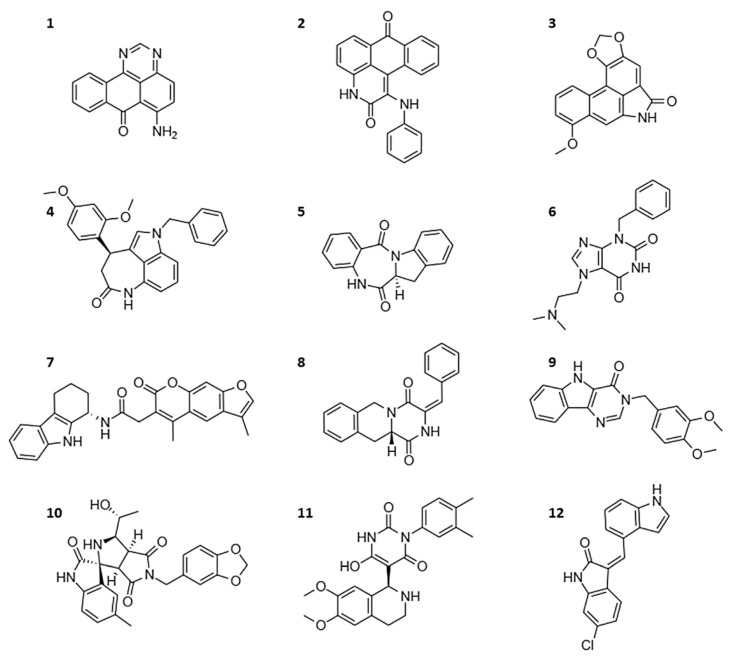
Chemical structures of *generation I* ligands selected from *in silico* screening for validation using *in vitro* GSK-3β binding assay experiments. The compounds chosen had diverse structural features: (**1**) 6-amino-7H-benzo[e]perimidin-7-one, (**2**) 1-(phenylamino)-3H-naphtho[1,2,3-de]quinoline-2,7-dione, (**3**) 8-methoxy-[1,3]dioxolo[4′,5′:4,5]benzo[1,2,3-cd]benzo[f]indol-5(6H)-one, (**4**) (R)-1-benzyl-3-(2,4-dimethoxyphenyl)-1,3,4,6-tetrahydro-5H-azepino[4,3,2-cd]indol-5-one, (**5**) (S)-12a,13-dihydro-6H-benzo[5,6][1,4]diazepino[1,2-a]indole-6,12(11H)-dione, (**6**) 3-benzyl-7-(2-(dimethylamino)ethyl)-3,7-dihydro-1H-purine-2,6-dione, (**7**) (S)-2-(3,5-dimethyl-7-oxo-7H-furo[3,2-g]chromen-6-yl)-N-(2,3,4,9-tetrahydro-1H-carbazol-1-yl)acetamide, (**8**) (S,E)-3-benzylidene-2,3,11,11a-tetrahydro-4H-pyrazino[1,2-b]isoquinoline-1,4(6H)-dione, (**9**) 3-(3,4-dimethoxybenzyl)-3,5-dihydro-4H-pyrimido[5,4-b]indol-4-one, (**10**) (3S,3′R,3a′S,6a′R)-5′-(benzo[d][1,3]dioxol-5-ylmethyl)-3′-((R)-1-hydroxyethyl)-5-methyl-2′,3′,3a′,6a′-tetrahydro-4′H-spiro[indoline-3,1′-pyrrolo[3,4-c]pyrrole]-2,4′,6′(5′H)-trione, (**11**) (S)-5-(6,7-dimethoxy-1,2,3,4-tetrahydroisoquinolin-1-yl)-3-(3,4-dimethylphenyl)-6-hydroxypyrimidine-2,4(1H,3H)-dione, and (**12**) (Z)-3-((1H-indol-4-yl)methylene)-6-chloroindolin-2-one.

On the basis of the benchmarking studies, the validated screening ***protocol 3*** was applied for virtual screening of the biogenic subset of the ZINC15 database using the workflow as shown in Scheme 1. The top-ranked 200 ligands (top-ranked 0.3%) from docking of 73,644 ligands were reviewed in terms of their protein–ligand interactions, but also ligand structural novelty (visual inspection). Twelve structurally-diverse ligands were selected to be validated as true inhibitors, using GSK-3β *in vitro* binding assay experiments. This *generation I* set of compounds explored a range of diverse chemical scaffolds (Figure 3). Table 2 shows the compounds’ Glide docking scores, QikProp results for CNS-related properties, and the results from the binding assay experiments. For the binding assays, compounds were initially screened for GSK-3β inhibition at 50 µM concentrations. Discarding the false-positive predictions, those compounds with >40% inhibition at this concentration (four of the twelve selected candidates, compounds **1**–**4**) were further tested to determine the IC_50_ values.

**Figure 4:**
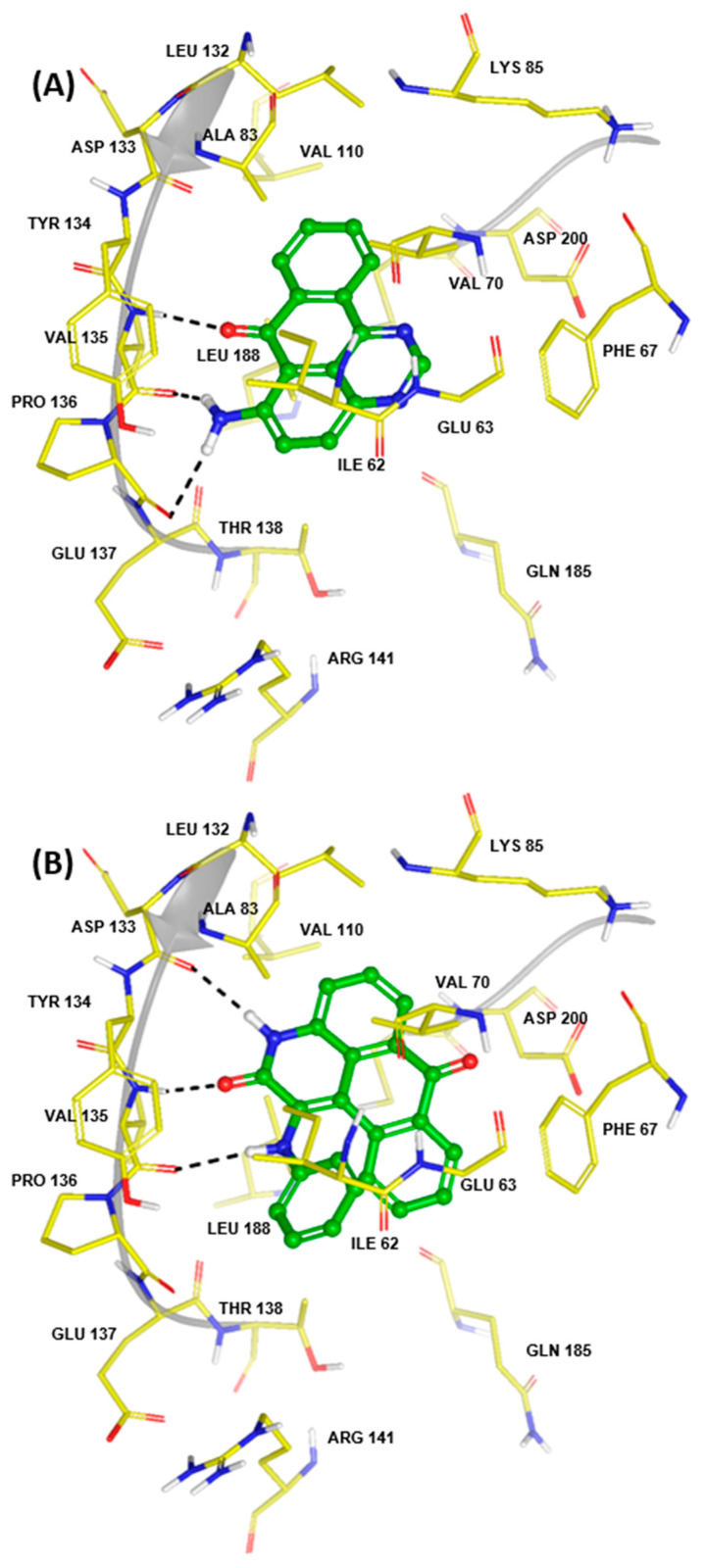
Predicted Glide-SP docking poses of the generation I hit compounds **1** (**A**) and **2** (**B**) from initial *in silico* screening of the biogenic database of ZINC15. Hydrogen bonds are shown as dashed black lines.

**Figure 5:**
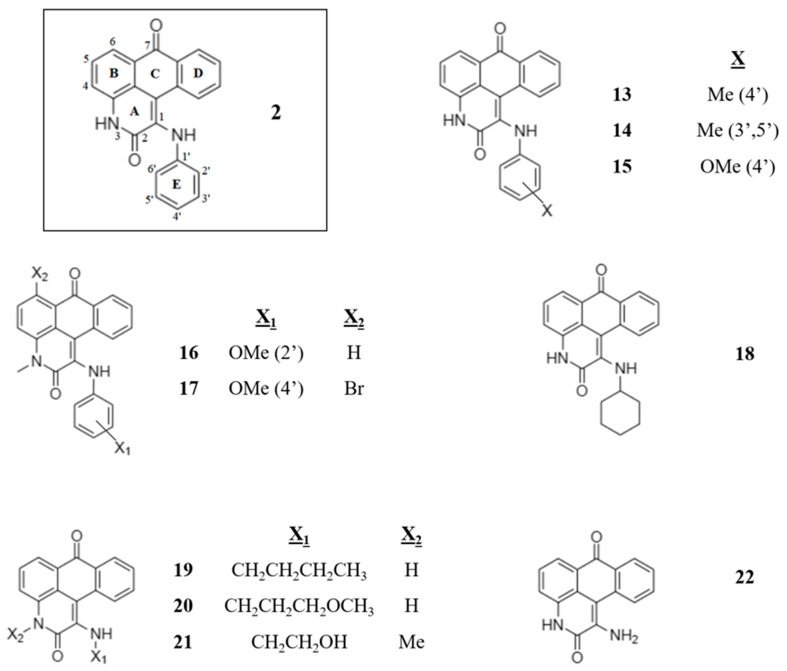
The 10 *generation II* analogues of hit compound **2** selected for GSK-3β *in vitro* binding assay experiments and structure activity relationship (SAR) analysis. The atoms and rings are labelled in **2** for discussion in the text.

Compounds **3** and **4**, with [1,3]dioxolo[4′,5′:4,5]benzo[1,2,3-cd]benzo[f]indol-5(6H)-one and (R)-1-benzyl-3-(phenyl)-1,3,4,6-tetrahydro-5H-azepino[4,3,2-cd]indol-5-one type scaffolds, respectively, were revealed as modest inhibitors, with IC_50_s of 63.73 and 87.51 µM, respectively. The predicted binding of these compounds is shown in Figure S1. However, compounds **1** and **2** were identified as potent hit compounds around the low micromolar range. Compound **1** had an IC_50_ value (1.63 µM), and its predicted binding can be seen in Figure 4A. The compound is predicted to exploit three hydrogen bonds near the hinge region involving the ligand carbonyl with Val135 NH, and ligand amine group with both Val135 O and Pro136 O. Ligand non-polar atoms are surrounded by non-polar residues that include Ile62, Phe67, Val70, Ala83, Val110, Leu132, and Leu188, although the smaller size of the compound means that some of these contacts are less than optimal. This 6-amino-7H-benzo[e]perimidin-7-one scaffold, while interesting, only partially occupies the binding site compared to the other potent hit compound **2** (IC_50_ = 20.55 µM). Changes in ligand solvent accessible surface area, ΔSASA, of ligands **1** and **2** on binding were 334.6 and 457.0 Å^2^, respectively; and for protein, 186.3 and 286.2 Å^2^, respectively. Compound **2**, a 1-(phenylamino)-3H-naphtho[1,2,3-de]quinoline-2,7-dione, was considered a more interesting scaffold for kinase inhibition and selectivity in this particular study, due to the better binding site occupancy/shape complementarity [30] and its protein–ligand interactions. Predicted binding of **2** from calculations is shown in Figure 4B. The docking output revealed the formation of three strong hydrogen bonds in the hinge region with Asp133 and Val135 backbones, which would make a significant contribution to the observed potency: ligand N(3)H (*c.f.* atom numbering and ring labelling scheme in Figure 5) with Asp133 O; C(2)O with Val135 NH; and N(C1)H with Val135 O. The ligand C(7)O in the back of the pocket does not form any direct interactions but may have some water-bridging potential [31], possibly with Asp200 sidechain carboxylate. Water-bridging in protein–ligand complexes can play a significant role in drug design [32]. Elsewhere, there is potential for van der Waals contacts with the same non-polar residues as mentioned for compound **1** above; the phenylamino moiety (ring E) points more in the direction of Ile62, but is also in reasonable proximity to the sidechain of Tyr134 from the hinge region, as well as Phe67 and Val70.

It was also necessary to consider that compound **2** has a 2-pyridone moiety (**t1**) with the potential to exist in a second 2-hydroxypyridine tautomeric state **t2** (lactam–lactim type tautomerism), as shown in Figure 5. 2-pyridone is known to be the predominant tautomer [33], but to provide reassurance that the predicted bound tautomeric **t1** state is also the most stable unbound state in the 1-(phenylamino)-3H-naphtho[1,2,3-de]quinoline-2,7-dione hit scaffold, Monte Carlo conformational searches were performed on both tautomers **t1** and **t2** of compound **2**, followed by DFT optimizations (gas phase) on the output conformations. Single-point solution phase energy calculations were then performed on the optimized conformations. The M06-2X/6-31+G* level of theory was used throughout due to its successful application in similar previous studies [34,35], as well as the recent notable performance of M06-2X for accurate calculation of relative tautomeric energies when comparing different semi-empirical and QM methods [36]. Water solvation effects were included using the SM8 continuum model [37]. The resulting most stable conformations of **t1** and **t2** are shown in Table 3, together with their relative energies. The most stable state in both gas and solution phases was **t1** by ~6–7 kcal/mol, in a conformation that was pleasingly similar to that predicted when bound to GSK-3β in the docked pose (RMSD heavy atoms = 0.138 Å). It should be noted, however, that both conformations for **t1** and **t2**, shown in Table 3, had an equivalent (symmetrically) stable conformation where the amino-phenyl group (ring E) was below the plane of the A-D ring system, as opposed to above. This feature becomes important in the binding of certain *generation II* analogues of compound **2**.

#### 2.2.2. *Generation II* Selection

All things considered, analogues of compound **2** were chosen for further studies; some had already been docked from the biogenic database, but others were based on additional commercial availability so as to facilitate a structure activity relationship (SAR) analysis. In total, 10 *generation II* analogues of compound **2** were selected (**13**–**22**, Figure 5) for a second round of *in vitro* GSK-3β binding assay experiments. The focus of the preliminary SAR was mainly substitutions on the phenylamino ring E (*c.f.* labelling scheme on **2**, Figure 5) or its replacement, together with determining the importance of certain hinge region hydrogen bonding. 3H-naphtho[1,2,3-de]quinoline-2,7-diones have been identified for kinase inhibition [38], but not the 1-(alkyl/arylamino)-3H-naphtho[1,2,3-de]quinoline-2,7-dione analogues, as explored here. The IC_50_s of all 10 *generation II* candidates were determined and are reported in Table 4.

From the *in vitro* binding assay data, we can see that all *generation II* compounds showed good inhibition (IC_50_ range 4.9–36.9 µM). The new compounds were predicted to bind in a similar orientation and position despite the different substitutions, with the docking poses for each compound included in the Supplementary Information (Figure S2). The most potent analogues were compounds **13**, **14**, **19** and **20**, all of which were at least 2 times more potent (IC_50_s < 10 µM) than the *generation I* hit compound **2**. The best compound, **19**, was >4 times more potent (IC_50_ = 4.9 µM).

**Figure 6:**
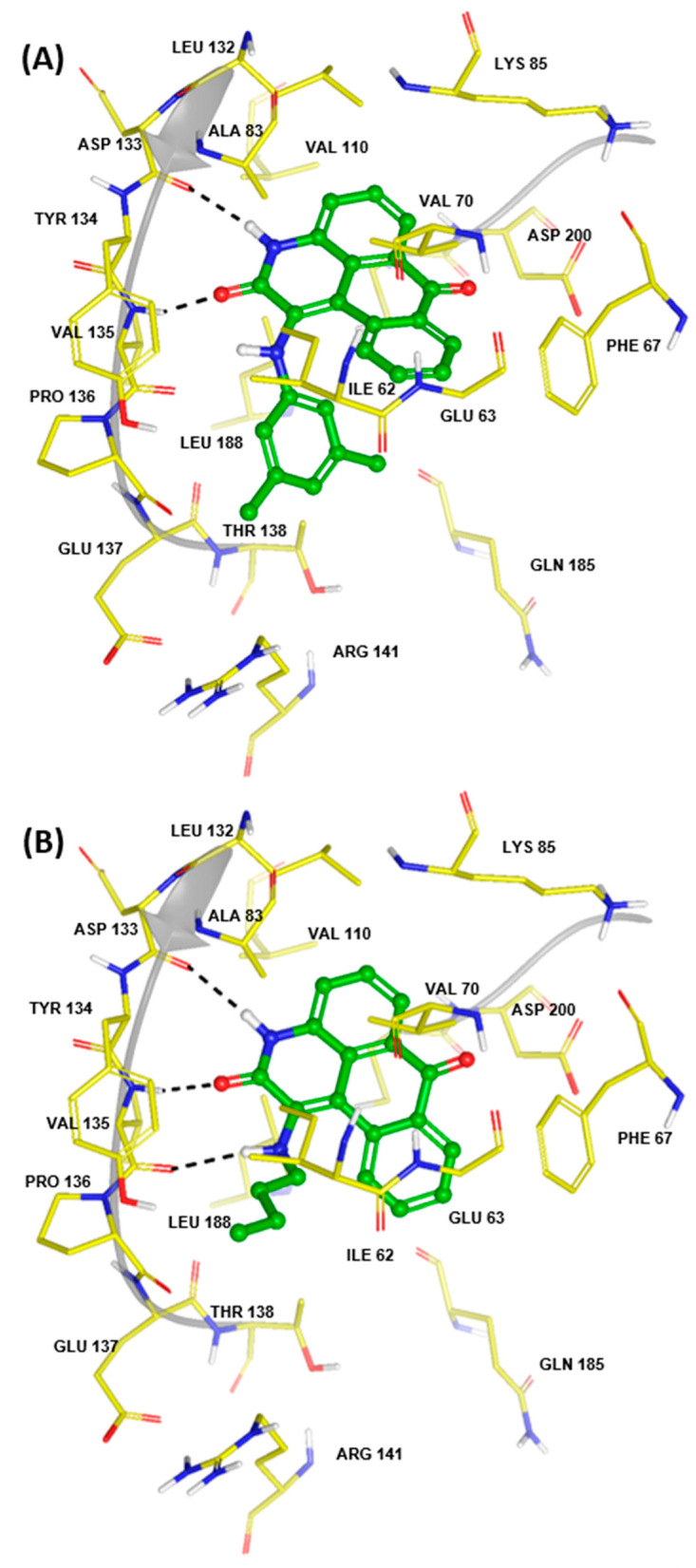
Predicted binding poses of two of the most potent generation II analogues, compounds (**A**) **14** (IC_50_ = 9.1 µM) and (**B**) **19** (IC_50_ = 4.9 µM). Hydrogen bonds are shown as dashed black lines.

Compounds **13**–**15** explored the effect of adding substituents on the phenylamino aromatic group (ring E) of the hit compound **2**. All substitutions revealed improved potencies (IC_50_s—9–11.5 µM); the 3′,5′-dimethyl substitution of the aminophenyl ring E for compound **14** produced the best IC_50_ of 9.1 µM, and its predicted binding to GSK-3β is shown in Figure 6A. The ligand exploits some similar protein–ligand interactions to the initial hit compound **2** in the hinge region: hydrogen bonding of N(3)H with Asp133 O, and C(2)O with Val135 NH. However, the aminophenyl ring E is now below the plane of the core rings A-D scaffold in the binding site (observed for all meta- or para-substituted ring E analogues), and, as a result, the expected third hinge region hydrogen bond of N(C1)H with Val135 O is slightly weakened (distance = 3.1 Å). This instead facilitates favourable contacts between the non-polar 3′,5′-dimethyl-phenyl ring E and residues such as Thr138 and Leu188.

Compounds **16** and **17** also had phenyl ring E substitutions, but additionally allowed us to probe the importance of N(3)H hydrogen bonding in the hinge region, with both compounds instead having an N(3)Me group. This did result in a less potent inhibitor for compound **16** (IC_50_ = 27.1 µM), but compound **17** (IC_50_ = 14.0 µM), despite the loss of one hinge region hydrogen bond with Asp133 O, was still more potent than **2**. The potential effects of the Br substitution (position 6, ring B) of **17**, however, should be factored in and considered more closely in future SAR analysis; nevertheless, the ortho-methoxy ring E substituent of **16** was clearly unfavourable, and this was also reflected in the docking score.

Compound **18** measured the effect of replacement of the phenyl ring E of hit compound **2** with a non-planar cyclohexyl moiety. The replacement was not favourable, resulting in a less potent compound **18** (IC_50_ = 25.3 µM). However, compounds **19**–**21** instead measured the effect of replacement of phenyl ring E with straight chain analogues; this was found to be effective with IC_50_ values of 4.9 and 9.1 µM for **19** (butyl chain) and **20** (3-methoxypropyl chain), respectively. Compound **21** (2-hydroxyethyl chain) had additionally a N(3)Me (compared to N(3)H for **19**–**20**) that affected hinge region hydrogen bonding, and this resulted in a less potent analogue (IC_50_ = 17.0 µM). Compound **19** was the most potent *generation II* analogue studied, and its predicted binding is shown in Figure 6B. Compound **19** forms the three hinge region hydrogen bonds, as expected, with Asp133 O, and Val135 NH and O backbone atoms. The butyl chain is above the core ring A-D plane and in close proximity to Ile62 and Tyr134 sidechains, making favourable contacts. Finally, removal of the alkyl/aryl substituents of the amino group at position 1 completely (compound **22**) led to the lowest potency *generation II* analogue (IC_50_ = 36.7 µM), highlighting the importance of these groups in the ATP-binding site.

### 2.3. Kinase Selectivity Screening

**Figure 7:**
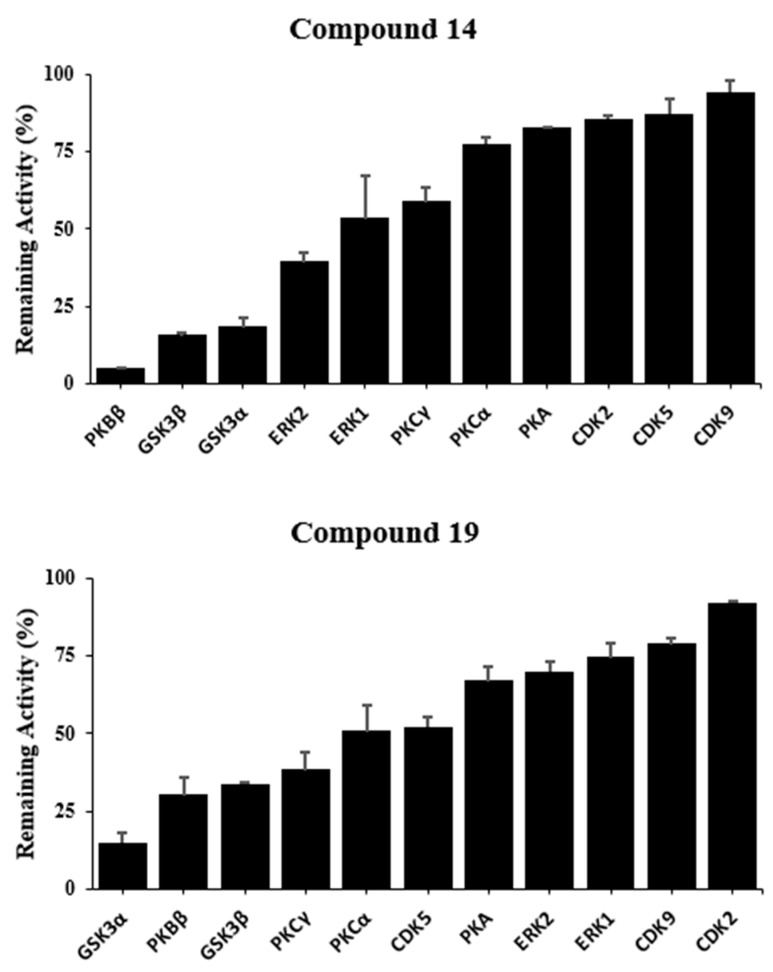
Single-concentration (50 μM) selectivity profiles of *generation II* compounds **14** and **19** versus 10 homologous kinases, shown as the % remaining activity ± standard deviation.

To probe the selectivity of two of the most potent identified inhibitors, **14** and **19** (one with alkyl chain and one with aryl group on N(C1)), selectivity screening against ten other kinases (CDK2, CDK5, CDK9, ERK1, ERK2, GSK-3α, PKA, PKBβ, PKCα, and PKCγ) was performed, with the results shown in Figure 7. The single-dose profiling (50 μM) revealed that, while there were four kinases (including GSK-3α and GSK-3β) inhibited with remaining activities < 50% for both compounds, there was generally good selectivity over the other kinases.

Additional IC_50_s for compounds **14** (against GSK-3α, PKBβ, and ERK2) and **19** (against GSK-3α, PKBβ, and PKCγ) were accordingly determined, for a more accurate measure of the relative potencies, with the results shown in Table 5. Both compounds potently inhibited GSK-3 isoforms with IC_50_s < 10 µM, and were in fact more potent for GSK-3α, with IC_50_s~2 µM. Compounds **14** (IC_50_ = 14.9 µM) and **19** (IC_50_ = 24.6 µM) also inhibited Protein Kinase B β (PKBβ, also known as AKT2) less potently. Inhibition of ERK2 was revealed for **14** (IC_50_ = 21.3 µM) and PKCγ in the case of **19** (IC_50_ = 11.0 µM). Interestingly, GSK-3 is a promiscuous substrate, and can be phosphorylated by several kinases including both PKB/Akt [39] and PKC [40], with activity inhibited through phosphorylation of Ser21 and Ser9 in GSK-3α and GSK-3β, respectively. In summary, achieving selectivity for ATP-binding site (type-I) inhibitors is challenging compared to, for example, type-II inhibitors that stabilize the inactive state [41,42]. However, both compounds **14** and **19** have unique selectivity profiles, and this polypharmacology can potentially be exploited to improve the therapeutic outcomes of kinase inhibitors [43].

### 2.4. ADME(T) Results & Analysis

ADME(T) property predictions for *generation I* and *II* compounds (**1**–**22**) were calculated using QikProp [28]. The results for log BB (logarithmic ratio between concentration of a compound in brain and blood) and polar surface area (PSA) parameters are presented in Table 2 (*generation I*) and Table 4 (*generation II*). These properties are good indicators of potential for blood–brain barrier (BB) permeability/CNS activity. Filters (MW < 450 Da [44,45], and QikProp CNS activity ≥ 0) were applied to the biogenic compound database during the *in silico* screening process (*c.f.* Computational Details section) to favour compounds that could be more easily developed into CNS-active drugs. For the four most potent identified *generation I* GSK-3β inhibitors (compounds **1**–**4**), the PSAs were in the range of 55.1–74.1 Å^2^. The PSA values were therefore all < 90 Å^2^, suggested by van der Waterbeemd et al. for potential CNS drugs [44]. With respect to log BB, a value of zero implies an equal concentration of inhibitor on either side of the blood brain barrier. The suggested log BB threshold value for compounds with CNS-active potential vary, but values as low as -1 have been suggested [46,47]. Compounds **1**–**4** are all above this threshold, with predicted log BB values ranging from −0.32 to −0.66.

Focussing on hit compound **2** and its 10 *generation II* analogues (Table 4), the PSAs and log BB values are again all within the aforementioned thresholds, with log BB values ranging from −0.21 to −0.85, and PSAs 69.0–89.4 Å^2^. The most potent compounds, **14** and **19**, have very similar predicted log BB values of −0.74 and −0.76, respectively; the PSA values for these compounds are likewise similar, at 74.5 Å^2^ and 73.0 Å^2^, respectively. Hence, these compounds have already predicted promising CNS-activity potential entering further lead optimization studies. In fact, the log BB and PSA values are relatively consistent with corresponding QikProp-predicted properties for a selection of 18 known CNS drugs (Table S3); close to half of the selected drugs calculated had log BB values in the range −1 to 0 (not taking into account any contribution of active transport). This highlights the applications of new property filtering tools for compound library design in virtual screening [48]. Furthermore, none of the *generation II* analogues displayed any violations of Lipinski’s ‘rule of five’ [49] or Jorgensen’s ‘rule of three’ [50,51] for oral bioavailability (Table S4). Running the compounds through the FAF-Drug4 server [52], which can reveal potential toxicity problems, all were predicted to be in the accepted category, meaning there were no structural alerts, and that they satisfied the physicochemical filter.

## 3. Conclusions

GSK-3 isoforms (α and β) are important targets for the development of new therapeutics, including against CNS disorders. In this study, with a focus on compounds with the potential for CNS-activity, a GSK-3β ATP-binding site virtual screening (docking) protocol was designed that led to the identification of two low micromolar hit scaffolds (compounds **1** and **2**) for kinase inhibitor development (*generation I* compounds), validated by *in vitro* GSK-3β binding assay experiments. The 6-amino-7H-benzo[e]perimidin-7-one scaffold of **1** (IC_50_ = 1.63 µM) will be explored in future work, but the focus of this study was 1-(phenylamino)-3H-naphtho[1,2,3-de]quinoline-2,7-dione (compound **2**, IC_50_ = 20.55 µM), due to its greater binding site occupancy and potential for kinase selectivity. A *generation II* set of 10 analogues of **2** was selected for a preliminary SAR evaluation. Four of these compounds (**13**, **14**, **19**, and **20**) revealed IC_50_s < 10 µM, and potential directions for further lead optimization. Compounds **13** and **14** highlighted the benefits of a substituted phenyl ring E, and **19** and **20** highlighted replacement of this ring with flexible hydrocarbon chains. Follow-up future SAR studies will help decipher other substitutions of importance. The most potent compounds, **14** (IC_50_ = 9.1 µM) and **19** (IC_50_ = 4.9 µM), demonstrated unique selectivity profiles following screening against 10 homologous kinases. Interestingly, both compounds were more potent for GSK-3α (IC_50_s~2 µM), and both inhibited PKBβ with IC_50_s of 14.9 µM (**14**) and 24.6 µM (**19**), while ERK2 inhibition was also important for **14** (IC_50_ = 21.3 µM) and PKCγ for **19** (IC_50_ = 11.0 µM). However, good selectivity for GSK-3α/β inhibition over the other kinases was observed, and, together with promising predicted CNS and oral bioavailability pharmacokinetic profiles, these compounds represent excellent candidates for experimental evaluation in cellular models of diseases such as Alzheimer’s disease and malignant brain tumours. The benefits of an *in silico*-motivated approach to the discovery phase of drug design has been emphasised.

## 4. Materials and Methods

### 4.1. Computational Details

#### 4.1.1. Ligand Preparation

To create active set ligands for the active/decoy benchmarking studies, ligands which had known IC_50_s ≤ 500 nM for GSK-3β inhibition were downloaded from the ChEMBL database (www.ebi.ac.uk/chembl (accessed on 1 April 2023)) [53]. The ligands were further filtered based on MW < 450 Da [44,45], and for a QikProp CNS activity ≥ 0. For the resulting 456 compounds, Canvas v 3.8 [28] was used for clustering and diversity selection of 50 representative active ligands. The clustering method used was ‘radial’ extended-connectivity fingerprints, with the directed sphere exclusion (DISE) method and Tanimoto similarity matrix for diversity selection. Using the 50 representatives, 3050 decoy ligands were generated using the DUD-e online decoy generator (http://dude.docking.org/generate (accessed on 1 April 2023)) [54]. Real inactive ligands were also selected from ChEMBL based on an IC_50_ of ≥ 100 µM, resulting in 144 extra compounds, so that the total benchmarking set consisted of 3244 compounds. The biogenics subset of the ZINC15 database (https://zinc15.docking.org/ (accessed on 1 April 2023)) [27] was filtered using the same MW < 450 Da and QikProp CNS ≥ 0 criteria, resulting in a set of 73,644 compounds that was used to screen for new inhibitor candidates. Compounds were prepared for docking using LigPrep v 5.6 [28] and a pH range of 7.0 ± 1.0.

#### 4.1.2. Protein Preparation

The solved crystal structure of GSK-3β in complex with bis-(indole)maleimide pyridinophane (PDB ID: 2OW3, resolution 2.80 Å) was downloaded from the Protein Databank (www.rcsb.org (accessed on 1 April 2023)) and prepared for calculations using Protein Preparation Wizard [28]. Bond orders were assigned and hydrogens added, with Prime v 6.2 selected to fill in any missing sidechains. Protonation states for basic and acidic residues were assigned using PROPKA [55], which calculated pK_a_s at pH = 7. The optimization of protein hydroxyl groups, histidine protonation states and potential side-chain C/N atom flips, and potential side-chain O/N atom flips of Asn and Gln residues were based on hydrogen bonding patterns. Finally, the system was gently minimised using OPLS3e forcefield [56], but with the RMSD (heavy atoms) kept within 0.3 Å of the original crystallographic positions. While water molecules within 5.0 Å of the native ligand were initially retained, these were removed for subsequent docking.

#### 4.1.3. Docking

Receptor grids for docking were created using Glide v 9.1 [28] using the prepared GSK-3β structure from PDB code: 2OW3. The shape and properties of the ATP-binding site were mapped onto a grid with dimensions 30 Å × 30 Å × 30 Å, centred on the native ligand (Figure 1). Hydrogen bond constraints were applied in the hinge region, so that at least 2 out of 3 hydrogen bonds would be formed with backbone Val135 O and NH, and Asp133 O. Calculations were performed *with* (***protocols 2*** and ***3***) and *without* these constraints (***protocol 1***). Standard parameters were otherwise used, and included default atomic charges and van der Waals scaling (0.8) for nonpolar ligand atoms to include modest induced-fit effects. Docking was performed in SP mode, with post-docking minimization and strain correction. Statistical analysis of docking performance for the benchmarking active/decoy set considered the following parameters. A receiver operator characteristic (ROC) plot shows the relationship between the true positive rate (TPR, or sensitivity) and the false positive rate (FPR, or specificity). The Area Under the ROC curve (AU-ROC) is a common way to summarize the performance in this plot and was calculated using Equations (1) and (2):

(1) $$U=\sum_{i=1}^{n}r_{i}-\frac{n\left(n+1\right)}{2}$$

where *U* is the Mann–Whitney–Wilcoxon *U* statistic, *n* is the total number of actives in the database, and *r_i_* is the rank of the *i*th active. Any unrecovered actives were given *r_i_* values at the end of the database. *U* was then used to calculate AU-ROC as follows:

(2) $$\mathrm{AU}\_\mathrm{ROC}=\frac{\left(n\times n_{D}\right)-U}{n\times n_{D}}$$

where *n_D_* is the total number of decoys and inactives. The range for AU-ROC values is 0–1, with 1 representing perfect performance and 0.5 meaning that the ranking is no better than random selection.

The Efficiency Factor (EF) was calculated as:

(3) $${\mathrm{EF}}_{x\%}=\frac{n_{x\%}/N_{x\%}}{n/N}$$

where *n*, as before, is the total number of actives, and *N* is the total number of compounds in database. The EF is described with respect to a given percentage of database ranks (top-1%, -2%, and -5% in this study), so that *n_x%_* represents the number of actives recovered in the top-*x*% of the ranked database containing *N_x_*_%_ compounds.

#### 4.1.4. Pharmacophore Modelling

Pharmacophore calculations were performed using Phase v 6.7 [28]. The models were created based on the 50 actives, but with the 143 real inactives employed to further test their quality. The hypothesised number of features was set at 3 and the scoring function set at the default phase hypo score. Conformations for each of the compounds were generated using ConfGen v 5.2 [28], with a maximum of 50 conformers per ligand saved. The survival score reflects how well the molecules are mapped onto the generated pharmacophore, as well as providing a general ranking of the hypotheses. An adjusted survival score (S_adj_) accounts for how well the models are able to discriminate between actives and inactives (survival score actives–survival score inactives).

#### 4.1.5. ADME(T) Calculations

Pharmacokinetics properties were calculated using QikProp v 6.8 (Schrodinger ref) in standard mode, and were used to consider properties of relevance to CNS-activity, as well as to predict oral bioavailability. The FAF-Drugs4 online server (https://fafdrugs4.rpbs.univ-paris-diderot.fr/links, accessed on 17 April 2023) [52] was also used, which considers potential toxicity problems that include structural alerts.

#### 4.1.6. DFT Calculations

To consider the relative stabilities of the free unbound ligand tautomeric states (**t1** and **t2**, Table 3) of compound **2**, DFT gas phase optimizations using Jaguar v 11.2 [28] were performed at the M06-2X/6-31+G* [57,58,59] level of theory. Frequency calculations were used to characterize the stationary points as true minima, as well as for calculation of the gas phase Gibbs free energies at 298.15 K. Single point energy calculations (M06-2X/6-31+G* + SM8) on the optimized geometries included the effects of water solvation using the SM8 model [37]. Input structures for these calculations came from Macromodel v 13.2 [28] conformational searches on **t1** and **t2** using the Monte Carlo Multiple Minima (MCMM) method. The conformational searches were 10,000 steps, with each step followed by a 100-step minimization using the truncated Newton conjugate gradient (TNCG) algorithm. The OPLS3e forcefield and analytical Generalized-Born/Surface-Area (GB/SA) water solvation model were employed. Default settings were otherwise employed.

### 4.2. Experimental In Vitro Binding Assays

All predicted inhibitors for testing (except compound **3**) were purchased from Vitas-M Laboratory, with purity ≥ 90%. Compound **3** was purchased from Biosynth Carbosynth with purity of 95%. Binding assay experiments against *Homo sapiens* GSK-3β were performed using a specialist service from the MRC Protein Phosphorylation & Ubiquitylation Unit at the University of Dundee (http://www.kinase-screen.mrc.ac.uk/ (accessed on 1 April 2023)). Initial assays underwent single concentration screening at 50 µM concentrations to determine those compounds showing the best inhibitory potential. Inhibitory activities were calculated based on maximal activities measured in the absence of an inhibitor. For selected candidates, the IC_50_ values were determined, defined as the concentration of a compound that reduces the enzymatic activity by 50% with respect to activity without inhibitors. In the kinase selectivity panel screening for **14** and **19**, the compounds were assayed at single concentration (50 μM) against CDK2, CDK5, CDK9, ERK1, ERK2, PKA, PKBβ, PKCα, PKCγ, GSK-3α, and GSK-3β. All binding assay experiments were performed in duplicate.

## Figures and Tables

**Table 1:** Enrichment statistical results for recognition of actives in the benchmarking studies using three different docking approaches. ***Protocol 1***: Glide-SP docking, without protein–ligand constraints. ***Protocol 2***: Glide-SP docking requiring at least 2 of 3 protein–ligand hydrogen bonds with hinge region backbone Asp133 O, Try134 NH, and O atoms. ***Protocol 3***: Glide-SP docking applying the hydrogen bond constraints from ***protocol 2***, combined with pre-docking pharmacophore filtering (three-point model shown in Figure 2).

Enrichment Statistics	*Protocol 1*	*Protocol 2*	*Protocol 3*
# Actives recovered ^a^	50	42	41
AU-ROC	0.8	0.76	0.77
**Top-ranked 1% Statistics**			
# Actives in top 1%	10	15	19
EF—1% ^b^	16	28	34
			
**Top-ranked 2% Statistics**			
# Actives in top 2%	16	16	22
EF—2% ^b^	15	16	22
			
**Top-ranked 5% Statistics**			
# Actives in top 5%	22	22	26
EF—5% ^b^	8.8	8.8	10

^a^ Total number (#) of actives was 50. ^b^ Enrichment factors (EF) can be at most 65 for 1% sampling, 50 for 2% sampling, and 20 for 5% sampling.

**Table 2:** Glide docking scores, CNS-relevant pharmacokinetics properties log BB, and ligand polar surface areas (PSA) calculated using QikProp, together with the experimental *in vitro* GSK-3β binding assay results for the 12 compounds in the *generation I* set (Figure 3).

Compound	Computational ^a^			Experimental
	Docking Score	Log BB	PSA (Å^2^)	IC_50_ (µM) *(% Inhibition at 50 µM)*
**1**	−8.21	−0.55	73.6	1.63 ± 0.30
**2**	−8.19	−0.66	74.1	20.55 ± 3.80
**3**	−8.84	−0.32	69.2	63.73 ± 3.3
**4**	−8.37	−0.41	55.1	87.51 ± 9.17
**5**	−8.29	−0.35	66.6	*(20%)*
**6**	−8.37	−0.29	89.7	*(11%* *)*
**7**	−8.31	−0.63	95.4	*(10%)*
**8**	−8.35	−0.50	66.0	*(10%)*
**9**	−8.41	−0.46	71.5	*(9%)*
**10**	−8.19	−0.63	138.7	*(6%)*
**11**	−8.92	−0.52	117.2	*(2%)*
**12**	−8.58	−0.30	54.5	*(NI) ^b^*

^a^ log BB, logarithmic ratio between concentration of a compound in brain and blood; PSA, polar surface area. ^b^ No inhibition.

**Table 3:** Relative energies and key geometry data for tautomers 1 (**t1**) and 2 (**t2**) of hit compound **2** following Monte Carlo conformational searches post-processed using DFT calculations.

**Skeletal Structure**	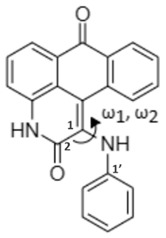	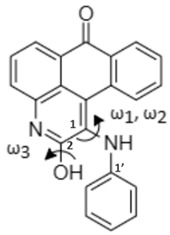
**Global minimum conformation**	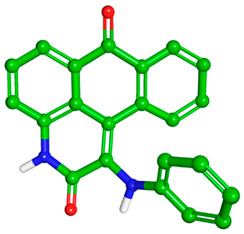	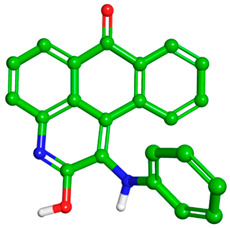
	**t1**	**t2**
**Energy (kcal/mol) ^a^**		
Relative GPE	0.0 (0.0)	7.1 (6.8)
Relative SPE	0.0	6.3
**Dihedral Angle (°) ^b^**		
*ω*_1_ [C(2)-C(1)-N-H]	9.3	15.7
*ω*_2_ [C(2)-C(1)-N-C(1′)]	−138.6	−128.9
*ω*_3_ [C(1)-C(2)-O-H]	-	−179.1

^a^ Energies are reported as relative gas phase (M06-2X/6-31+G*) energies (GPEs) and relative solution phase (M06-2X/6-31+G* + SM8) energies (SPEs); also shown in parentheses are the relative gas phase Gibbs energies at 298.15 K. ^b^ Key dihedral angles that demonstrate the deviation from planarity of the aminophenyl substituent at C1. For each tautomer, there is an equivalent global minimum conformation (same relative energies) where the aminophenyl group is below the plane of the other rings (as opposed to above) and the dihedral angles *ω*_1_, *ω*_2_ and *ω*_3_ have opposite sign.

**Table 4:** Glide docking scores, CNS-relevant pharmacokinetics properties log BB, and ligand polar surface areas (PSA) calculated using QikProp, together with the experimental *in vitro* GSK-3β binding assay results for the 10 *generation II* set of compounds, analogues of compound **2** (Figure 5). Corresponding data for **2** are shown for comparison.

Compound	Computational ^a^			Experimental
	Docking Score	Log BB	PSA (Å^2^)	IC_50_ (µM)
**2**	−8.19	−0.66	74.1	20.55 ± 3.80
**13**	−8.32	−0.70	74.1	9.4 ± 0.5
**14**	−8.44	−0.74	74.5	9.1 ± 0.2
**15**	−8.31	−0.76	82.4	11.4 ± 0.6
**16**	−4.79	−0.41	70.0	27.1 ± 1.1
**17**	−7.68	−0.21	69.0	14.0 ± 3.1
**18**	−8.41	−0.60	73.3	25.3 ± 3.5
**19**	−7.54	−0.76	73.0	4.9 ± 0.2
**20**	−7.89	−0.83	81.8	9.1 ± 1.7
**21**	−7.91	−0.85	87.7	17.0 ± 2.7
**22**	−8.40	−0.85	89.4	36.7 ± 5.7

^a^ log BB, logarithmic ratio between concentration of a compound in brain and blood; PSA, polar surface area.

**Table 5:** Results of the IC_50_ determinations for compounds **14** and **19** against kinases for which there was <50% remaining activity in the single concentration selectivity profiles shown in Figure 7.

Kinase	Compound IC_50_ (µM)	
	14	19
GSK-3α	2.3 ± 0.4	1.7 ± 0.1
GSK-3β	9.1 ± 2.4	4.9 ± 0.2
PKBβ	14.9 ± 1.5	24.6 ± 0.2
ERK2	21. 3 ± 2.3	-
PKCγ	-	11.0 ± 1.5

## Data Availability

Data sharing not applicable.

## Supplementary Materials

The following supporting information can be downloaded at: https://www.mdpi.com/article/10.3390/ph16050661/s1, Supplementary Tables S1–S4 and Figures S1 and S2.
