# Supplementary material for: In Silico-Motivated Discovery of Novel Potent Glycogen Synthase-3 Inhibitors: 1-(Alkyl/arylamino)-3H-naphtho[1,2,3-de]quinoline-2,7-dione Identified as a Scaffold for Kinase Inhibitor Development
Source: Pharmaceuticals (Basel). 2023 Apr 28;16(5):661. doi: 10.3390/ph16050661 (PMC10223992; doi:10.3390/ph16050661)
Supplement: Supplementary file 1 [file pharmaceuticals-16-00661-s001.zip › pharmaceuticals-2352104-supplementary.pdf]

Supporting Information

In Silico-Motivated Discovery of Novel Potent Glycogen Synthase–3 inhibitors: 1-(Alkyl/arylamino)-3H-naphtho[1,2,3-de]quinoline-2,7-dione Identified as a Scaffold for Kinase Inhibitor Development

Thomas D. Emmerich and Joseph M. Hayes*

School of Pharmacy & Biomedical Sciences, University of Central Lancashire, Preston PR1 2HE, UK

* Corresponding authors
tel: +441772894334 (JMH); email: jhayes@uclan.ac.uk

1. Supplementary Information Tables

Table S1. The CheEMBL ID, IC_{50} data and SMILES for the 50 known diverse actives (IC_{50}s ≤ 500 nM) from the ChEMBL database used in the active/decoy benchmarking studies.

ChEMBL ID	IC_{50} (nM)	SMILES
CHEMBL1254896	53	Cc1[nH]c2ccccc2c1C1=C(NCCc2ccc(F)cc2)C(=O)N(C)C1=O
CHEMBL1271441	100	NC(=O)c1cc(-c2ccncc2F)[nH]c1-c1ccccc1
CHEMBL160585	187	COc1ccccc1C1=C(N2CCc3ccccc32)C(=O)NC1=O
CHEMBL1630434	10.8	Oc1ccc(Nc2ncnc3[nH]cnc23)cc1CN(CCCl)CCCl
CHEMBL1630451	325	COc1ccc(-c2cc(-c3ccc(F)cc3)[nH]c(=O)c2O)cc1
CHEMBL1630483	53	COc1ccc(Nc2nnc(-c3ccc(C(F)(F)F)cc3)o2)cc1
CHEMBL1801628	500	Nc1c(Br)cc(C(=O)C(Br)Br)cc1Br
CHEMBL182891	320	COc1ccc(-c2cc3c(NC(=O)CC4CCCC4)ncnc3o2)cc1
CHEMBL1830063	4	O=C1Nc2ccccc2/C1=C1/Nc2cc(Br)ccc2C1=O
CHEMBL1938959	490	Cc1ccccc1-n1cc(-c2ccccn2)nn1
CHEMBL1957231	5.012	COc1ccc(-c2ocnc2C(=O)NCc2ccn3ccnc3c2)cc1Cl
CHEMBL1969664	31	N#Cc1ccc2[nH]c(O)c(-c3ccc(CN4CCOCC4)cn3)c2c1
CHEMBL2022413	300	Br.OCC/N=c1\nc(-c2ccccc2)n(-c2cccc3ccccc23)s1
CHEMBL2024372	500	Br.CCOC(=O)C/N=c1\nc(-c2ccc(OC)cc2)n(-c2ccccc2)s1
CHEMBL2048673	9	O=C(NCCc1cccnc1Cl)c1ccnc2[nH]c(-c3ccoc3)nc12
CHEMBL2064628	227	CN(C)C[C@@H](OC(=O)N1Cc2c(NC(=O)C(C)(C)C)n[nH]c2C1(C)C)c1ccccc1
CHEMBL2094331	4.19	O=C1Nc2ccccc2/C1=C/c1ccccn1
CHEMBL215803	13	C[C@@H]1C[C@H]2CN1CCn1nc3c(cccc3c1O)-c1nc3c(cccc3nc1O)O2
CHEMBL2297166	120	Cc1ccc(-c2c[nH]c(C3COCCN3Cc3c[nH]c4ccccc34)n2)cc1
CHEMBL254582	200	Cc1cccc(N2CCN(CCCNC(=O)c3nc(-c4cccnc4)no3)C(C)C2)c1
CHEMBL259271	5	CCN1CCN(CCCCC(=O)Nc2n[nH]c3nnc(-c4ccccc4)cc23)CC1
CHEMBL270473	1.5	Cc1cc(-c2cc(-c3nc4ccc(CN5CCN(C)CC5)cc4[nH]3)c(=O)[nH]n2)cc(C)c1O
CHEMBL303321	6.31	COc1cccc(-n2ncc3c(N/N=C/c4ccc(OCCN(C)C)cc4)ncnc32)c1
CHEMBL3110144	240	Cc1nn(-c2ccc(Cl)cc2Cl)c2c1c1c(c3cccn32)C(=O)NC1=O
CHEMBL317657	14	O=C(Nc1n[nH]c2nc(-c3ccco3)c(Br)cc12)C1CCN(Cc2ccccc2)C1
CHEMBL328194	75	COc1cc2c(cc1OC)-c1[nH]c3ccc(C(F)(F)F)cc3c1CC(=O)N2
CHEMBL357136	500	O=C(CCl)c1scc(Br)c1Br
CHEMBL361567	80	COc1ccc(-c2oc3ncnc(N)c3c2-c2cccnc2)cc1
CHEMBL362155	50.12	FC(F)(F)c1cccc(-c2nn3ncccc3c2-c2ccnc(Nc3ccc4c(c3)OCCO4)n2)c1
CHEMBL3661029	50	Fc1cnc2[nH]nc(Nc3cc(C(F)(F)F)cc(-c4ccccc4Cl)n3)c2c1
CHEMBL3735603	20	COc1ccncc1-c1ccc2[nH]nc(C(=O)NCC3CCN(CCc4ccccc4)CC3)c2c1
CHEMBL3735890	6.1	COc1ccc2c(c1C(C)N1CCNCC1)O/C(=C\c1n[nH]c3ncccc13)C2=O
CHEMBL3891139	46	O=C(/C=C/c1cnc2[nH]cc(-c3ccccc3)c2c1)NCCN1CCOCC1
CHEMBL398621	35	Clc1ccccc1-c1cc(NCOc2cccnc2)n2ncc(Br)c2n1
CHEMBL403405	30	COc1cccc(Nc2nccc(C3=C(c4ccccc4)NN4C=CC=CC34)n2)c1
CHEMBL4071429	1.7	COc1cc(F)cc(F)c1C1CN(c2nc(-c3ccncn3)cc(=O)n2C)CCN1.Cl
CHEMBL4077172	5	O=C1Cc2cc(Br)ccc2/C1=c1/[nH]c2ccccc2/c1=C\O
CHEMBL4162850	1.3	O=CN1Cc2cccc3c(C4=C(c5cnc6ccccn56)C(=O)NC4=O)cn(c23)CC1N1CCOCC1
CHEMBL445813	200	O=C(NC1CCNCC1)c1n[nH]cc1NC(=O)c1c(Cl)cccc1Cl
CHEMBL4568120	218	C=CCOC(=O)c1cc(-c2ccc(/C=C3/NC(=S)NC3=O)o2)ccc1Cl
CHEMBL4592870	471	COc1cccc(CCC(=O)Nc2cc(-c3[nH]c(SC)nc3-c3ccc(F)cc3)ccn2)c1
CHEMBL461139	300	O=c1[nH]cnc2[nH]c(-c3ccnc(/C=C/c4ccc(CN5CCOCC5)cc4)c3)cc12
CHEMBL4635819	82	Cc1ccc(-n2cc(CSC3=NC(c4ccccc4)CC(=O)N3C)nn2)cc1C
CHEMBL4643895	390	CCc1ccc(-n2cc(CSc3nnc(Cn4c(C(F)(F)F)nc5ccccc54)o3)nn2)cc1
CHEMBL484685	16.1	Cn1cc(C2=C(c3coc4ccc(F)cc34)C(=O)NC2=O)c2cc(C#CC3CC3)ccc21
CHEMBL495039	35	COc1cc(NCCCN)c2nccc(C)c2c1Oc1cccc(C(F)(F)F)c1
CHEMBL513570	2.5	COc1ccc(-n2cnc3ccc(-c4nnc(SCc5cccc(C(F)(F)F)c5)o4)cc32)cc1
CHEMBL564450	0.14	COc1cc(C2=C(c3cn(CCN4CCN(C)CC4)c4ccccc34)C(=O)NC2=O)c2occc2c1

CHEMBL570580	92	CCc1cc2c(c3cc(OC)c(OC)cc13)C(=O)NC2=O
CHEMBL575882	190	CC[S@@+]([O-])c1ccc(-c2coc3ccc(-c4ccc(C)o4)cc23)cc1

Table S2. Three-point pharmacophore hypothes results generated using the program Phase v6.7. The ADR *hypothesis 4* pharmacophore (Figure 2) was chosen due to the requirement for a filter containing both the hydrogen bond acceptor (A) and donor (D) features for hinge region binding. *Hypothesis 4* was the best of the A and D feature pharmacophores through its S_{adj} score (2.470) and had the highest number of matched actives (40) for all hypotheses; the hypothesis identified an aromatic ring group (R) as the third important binding feature for actives.

Hypothesis	Features[a]	Survival Score	Survival Inactives	S_{adj} [b]	# Matches [c]
1	DRR	4.321	1.785	2.536	35
2	DRR	4.282	1.863	2.419	31
3	DRR	4.250	1.719	2.531	30
**4**	**ADR**	**4.043**	**1.573**	**2.470**	**40**
5	DRR	4.307	1.908	2.399	35
6	ADR	4.040	1.595	2.445	33
7	ADR	3.932	1.484	2.448	29

[a] D = H-bond donor group, R = Aromatic Ring group, and A = H-bond acceptor group. [b] Adjusted survival score (survival score – survival inactives). [c] The total number of surviving actives from the initial 50.

Table S3. QikProp v6.8 calculated CNS-activity relevant properties MW, CNS activity score, log BB and polar surface area (PSA) and for a selection of eighteen drugs currently used to treat different CNS conditions.

Drug	MW (Da)	CNS	log BB	PSA (Å^2)
**Carbamazepine**	265.4	2	0.81	18.7
**Clonazepam**	315.7	-2	-1.08	102.2
**Codeine**	299.3	2	0.45	43.5
**Dextroamphetamine**	135.2	1	0.42	25.0
**Diazepam**	284.7	1	0.20	47.7
**Donepezil**	236.3	0	-0.19	51.6
**Eszopiclone**	388.8	1	-0.14	109.0
**Lisdexamfetamine**	263.3	-1	-0.72	86.4
**Memantine**	379.5	1	0.10	50.1
**Mephobarbital**	246.2	0	-0.39	86.2
**Methadone**	295.4	1	0.35	25.4
**Methylphenidate**	233.3	1	0.30	46.6
**Mirtazapine**	179.3	2	0.54	25.4
**Morphine**	285.3	1	0.05	57.9
**Naloxone**	327.3	1	-0.32	79.5
**Pentobarbital**	226.3	0	-0.63	97.4
**Phenobarbital**	232.2	-1	-0.55	100.8
**Zolpidem**	307.3	1	0.04	42.0

Table S4. Results of ADME property predictions for the *generation II* compounds **13-22**, with data for hit compound **2** shown for comparison.[a]

Ligand	Lipinskis's Rule of Five & Violations (V)[b]					Jorgensen's Rule of Three & Violations (V)[b]				PSA [Å^2][c]	log K_{hsa}[d]	log *BB*[e]
	M_r [Da] (<500)	HBD[f] (≤5)	HBA[g] (≤10)	log $P_{(o/w)}$ (<5)	V	Caco-2 [nm s^{-1}][h] (>22)	log *S* (>-5.7)	NPM[i] (<7)	V			
**2**	338.365	2	5	2.937	0	680.758	-4.145	2	0	74.1	0.296	-0.66
**13**	352.392	2	5	3.349	0	602.625	-5.083	2	0	74.1	0.45	-0.70
**14**	366.418	2	5	3.58	0	605.578	-5.334	4	0	74.5	0.576	-0.74
**15**	368.391	2	5.75	3.144	0	635.775	-4.639	3	0	82.4	0.316	-0.76
**16**	382.418	1	6.25	3.578	0	779.054	-4.85	4	0	70.0	0.362	-0.41
**17**	461.314	1	6.25	4.05	0	1563.995	-5.171	2	0	69.0	0.46	-0.21
**18**	344.412	2	5.5	2.977	0	689.726	-4.594	1	0	73.3	0.343	-0.60
**19**	318.374	2	5.5	2.731	0	634.768	-4.093	2	0	73.0	0.152	-0.76
**20**	334.374	2	7.2	2.173	0	686.733	-3.609	3	0	81.8	-0.138	-0.83
**21**	320.347	2	7.7	1.547	0	388.773	-3.171	3	0	87.7	-0.288	-0.85
**22**	262.267	2.5	5.5	1.033	0	267.379	-2.847	1	0	89.4	-0.263	-0.85
**Range[j]**	**130-725**	**0-6**	**02-20**	**-2-6.5**	**-**	**<25 poor; > 500 great**	**-6.5-0.5**	**1-8**	**-**	**7-200**	**-1.5-1.5**	**-3.0-1.2**

[a] ADME data were calculated as described in the text using Qikprop. [b] Rules as listed in the columns, with number of violations given in the V column. [c] PSA represents the van der Waals (polar) surface areas of N and O atoms. [d] log K_{hsa}: predicted binding to human serum albumin. [e] log BB: the predicted blood-brain barrier coefficient. [f] Number of hydrogen bond donors. [g] Number of hydrogen bond acceptors. [h] Caco-2 cell permeability. [i] Number of primary metabolites. [j] Range for 95% of known drugs - reference: QikProp version 3.5 User's Manual.

2. Supplementary Information Figures

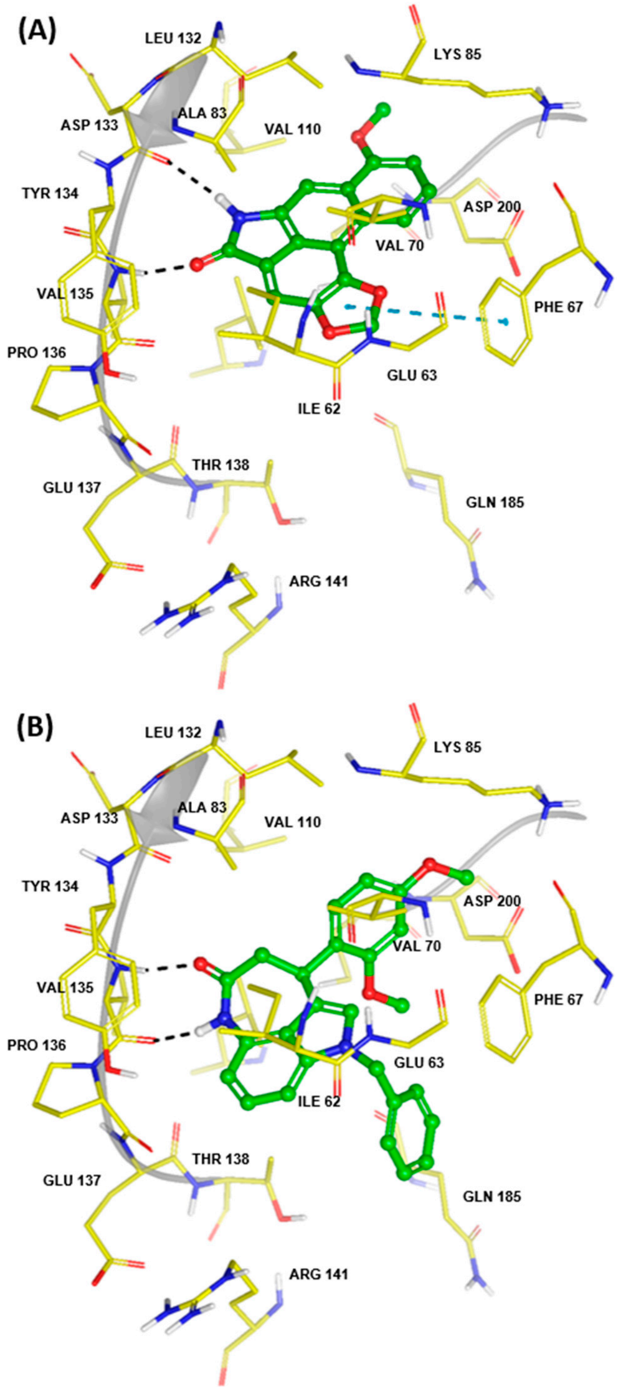

Figure S1. Predicted Glide-SP docking poses of the *generation I* compounds **3** (**A**) and **4** (**B**) from initial *in silico* screening of the biogenic database of ZINC15. Both compounds form hinge region hydrogen bonds with backbone Val135 NH and Asp133 O. For **3**, the dioxolo-ring is T-shaped with respect to the Phe67 side-chain phenyl, and for compound **4**, the phenyl group is close to forming parallel displaced π-π interactions with the same side-chain.

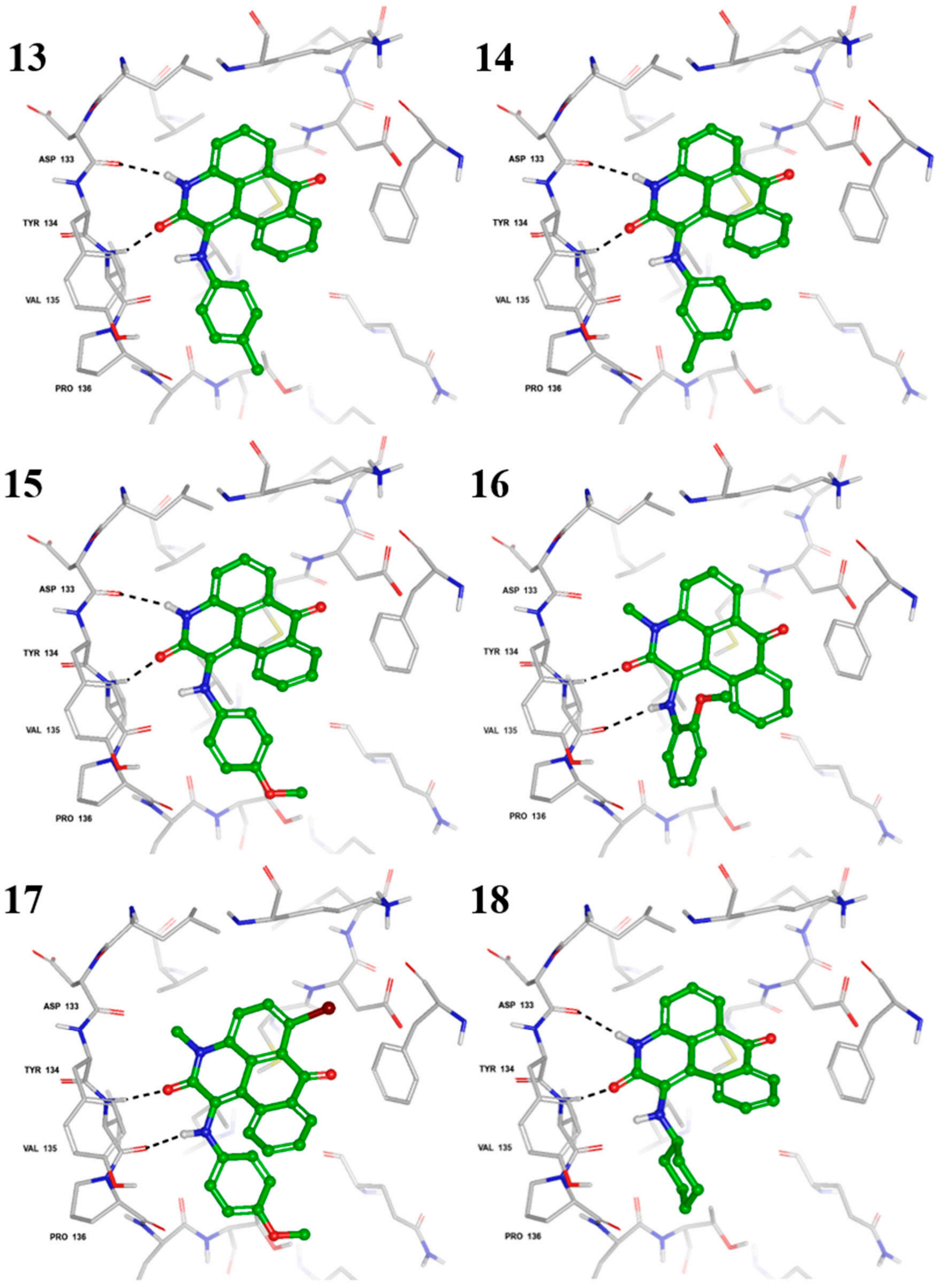

Figure S2. Predicted binding of the *generation II* set of 1-(alkyl/arylamino)-3H-naphtho[1,2,3-de]quinoline-2,7-dione analogues (**13–22**, Figure 5) of hit compound **2** from the Glide-SP docking.

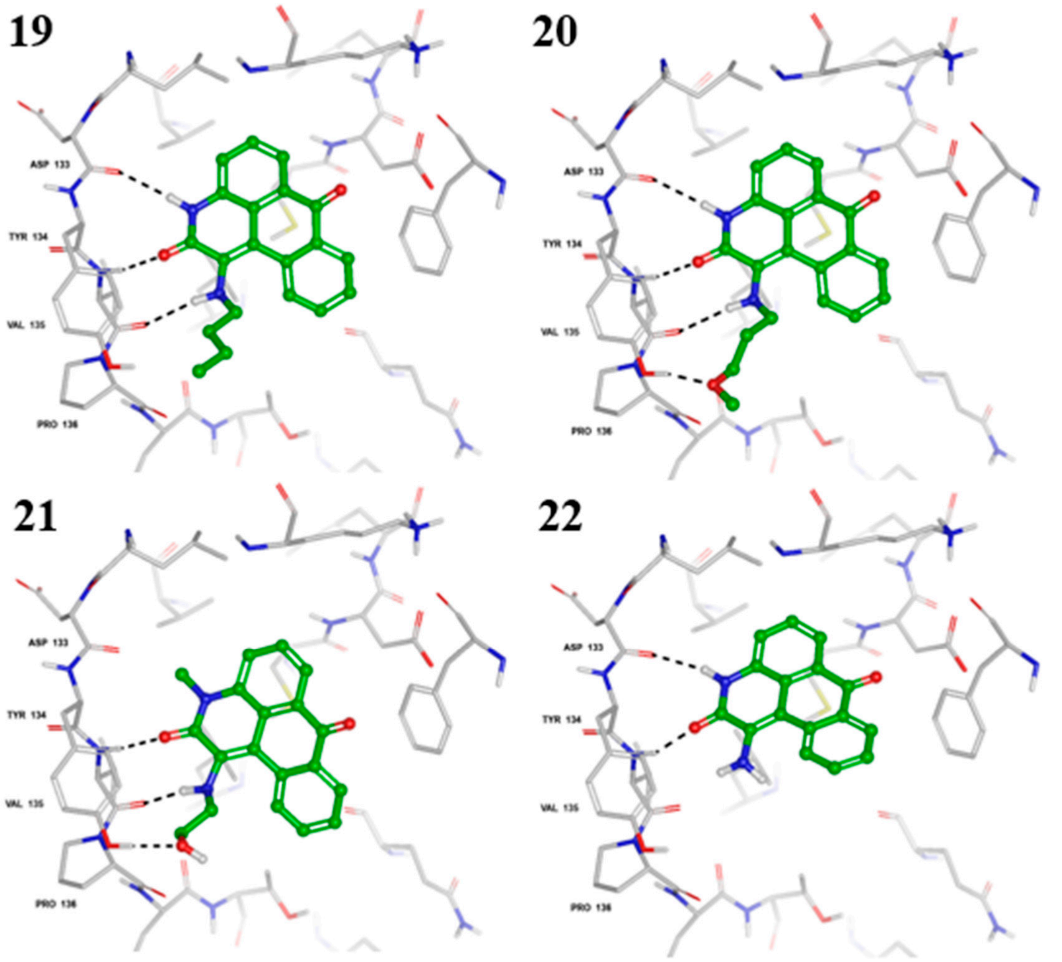

Figure S2 (continued). Predicted binding of the *generation II* set of 1-(alkyl/arylamino)-3H-naphtho[1,2,3-de]quinoline-2,7-dione analogues (**13–22**, Figure 5) of hit compound **2** from the Glide-SP docking.
